# A 10-Year Follow-Up Study of Social Ties and Functional Health among the Old: The AGES Project

**DOI:** 10.3390/ijerph14070717

**Published:** 2017-07-03

**Authors:** Chiyoe Murata, Tami Saito, Taishi Tsuji, Masashige Saito, Katsunori Kondo

**Affiliations:** 1 National Center for Geriatrics and Gerontology, 7-430 Morioka, Obu 474-8511, Japan; t-saito@ncgg.go.jp; 2 Center for Preventive Medical Sciences, Chiba University, 1-8-1 Inohana, Chuo-ku, Chiba 260-8670, Japan; tsuji.t@chiba-u.jp (T.T.); kkondo@chiba-u.jp (K.K.); 3 Department of Social Welfare, Nihon Fukushi University, Okuda, Mihamacho, Chita-gun, Aichi 470-3295, Japan; masa-s@n-fukushi.ac.jp

**Keywords:** social ties, social support, functional health

## Abstract

In Asian nations, family ties are considered important. However, it is not clear what happens among older people with no such ties. To investigate the association, we used longitudinal data from the Aichi Gerontological Evaluation Study (AGES) project. Functionally independent older people at baseline (*N* = 14,088) in 10 municipalities were followed from 2003 to 2013. Social ties were assessed by asking about their social support exchange with family, relatives, friends, or neighbors. Cox proportional hazard models were employed to investigate the association between social ties and the onset of functional disability adjusting for age, health status, and living arrangement. We found that social ties with co-residing family members, and those with friends or neighbors, independently protected functional health with hazard ratios of 0.81 and 0.85 among men. Among women, ties with friend or neighbors had a stronger effect on health compared to their male counterparts with a hazard ratio of 0.89. The fact that social ties with friends or neighbors are associated with a lower risk of functional decline, independent of family support, serves to underscore the importance of promoting social ties, especially among those lacking family ties.

## 1. Introduction

The association between social relationships and health has been extensively investigated. Social networks, and the degree to which individuals are embedded in supportive social relationships, are related to favorable health outcomes. For example, those with richer social networks have a lower risk of mortality [1], morbidity [2], and even functional decline [3,4,5,6]. The psychological effects of social relations are also documented. Especially among older persons, informal ties such as with family members, friends, neighbors, or relatives, are important sources of well-being [1]. In studies of social relationships and health, various measurement strategies have been used for assessing social relationships. One strategy was a quantity-based approach (i.e., number of social ties or participation in organizations), and the other was a quality-based approach (i.e., function or nature of social support). When one person interacts with another, something is exchanged. Social support refers to that something exchanged between persons. In other words, social support is embedded in people’s social networks [1]. Social support is exchanged as a form of daily assistance, care, financial assistance, gift giving, counseling, or emotional assurance.

However, there are relatively few studies on the longitudinal effects of social support on health. Studies in the US demonstrated that instrumental support was associated with higher disability risk [7,8]. Cross-sectional studies in China demonstrated a stronger association of support from spouses with well-being compared to support from children or others [9,10]. A similar result has been reported in South Korea [11]. These studies have attempted grouping social network types based on marital status, number of friends, number of children, or group participation, and found that a diverse network, characterized by the presence of both family and friends, was more beneficial for health, and a restricted network with only limited family ties was related to poor health [11,12,13]. Although few studies assess the effect of social support on health in Asia, a study in South Korea demonstrated the longitudinal effect of social network changes on mental health [14]. Recent Japanese studies demonstrated the protective effect of social participation on functional health among the old [4,5,6]. However, in Asia, the longitudinal effect of social ties on disability risk remains unclear.

Cultural differences are also implicated in the association between social support and health. Cheng et al. suggested that interactions with friends are less well-being-enhancing in Asian societies than those in Western societies. This is partly explained by the relative importance of the norms of reciprocity and social harmony in Asian cultures. In such societies, the cost of seeking help from others, especially from non-family members, might be higher compared to more individualistic Western societies. In fact, the advantage observed in Western studies of non-family networks for health benefits was not detected in this study in China [13]. In a cross-sectional study in South Korea [11], a couple-focused network was associated with better mental health. In addition, they found more older persons were isolated compared to those in China [13] or in Japan [15]. In general, in studies in Asian nations, friends or neighbors were not considered to be sources of support when needs arose [16].

However, recent studies in Japan suggested a health protective effect from support exchange with outside family members among the old [9,10]. Due to a rapid decline in traditional family systems, the number of older persons living alone is on the rise in Asian nations. Japan is no exception. Household size is getting smaller, as in other industrialized nations, and more adult children live separately from their parents once they get married [16,17]. According to the annual report released by the Cabinet Office of Japan for the fiscal year 2014, 23.3% of households with persons aged 65 and over were one-person households [18]. This might lead to a shifting importance toward more diverse social networks including non-family members among the old. Thus, we expect that, in present day Japan, the health protective effect from non-family ties would be observed independent of family ties. Since the longitudinal effect of support from non-family members remains unclear, we attempt to fill the knowledge gap by assessing this effect, using public insurance data maintained by local governments in Japan.

In this study, we analyzed the association between social ties, assessed by social support exchange and onset of functional disability, using 10-year follow-up data. Our aim was to investigate the relative effect of ties with family or relatives (co-residing, or living apart) compared to ties with non-family members (friends or neighbors) on the functional health of older persons in Japan.

## 2. Materials and Methods

### 2.1. Data and Participants

The present study is a part of the Aichi Gerontological Evaluation Study (AGES) Project. This is a community-based prospective cohort study in Japan in which investigators evaluated factors associated with incident functional disability among non-institutionalized older people aged 65 years or above. The baseline survey was conducted in October of 2003. Questionnaires were sent to a random sampling of community-living older adults aged 65 years or older in six large municipalities and a complete census from four smaller cities. A detailed description of the study population and the baseline survey has been published [19]. Study participants were comparable to entire older Japanese populations in terms of age and sex. Detailed descriptions of questions on the survey were also published [20].

After excluding those with incomplete data on sex and age, 15,313 people (7381 men and 7932 women) were introduced into the cohort and followed for 10 years from 1 November 2003 to 28 March 2013, using the Long-term Care Insurance (LTCI) system database maintained by local governments. Japan’s LTCI system is a government-operated national insurance system for long-term care and was introduced in April of 2000, to allow every Japanese person aged 65 and older with functional limitations or dementia access to care for basic activities of daily living [21]. Since receipt of benefits under this LTCI system is on an application basis, some people do not receive benefits despite being dependent for activities of daily living for various reasons, such as the availability of family members to provide care or financial burden, since a 10% co-insurance is required to use services under the LTCI. Thus, we asked about basic activities of daily living, such as using the toilet, bathing, or transferring, in the survey of 2003 to eliminate those already functionally dependent as a baseline. Those with missing data in their activities of daily living were also eliminated. This procedure left 14,088 older people or, 92% of the total sample of this cohort for the analyses. The study protocol and informed consent procedure were approved by the Ethics Committee in Research of Human Subjects at Nihon Fukushi University (#10-05).

### 2.2. Incident Disability

We obtained information regarding certification of long-term care needs, death, and relocation of participants (e.g., moving out of the study area) from the LTCI system database. We defined people with functional disability as those who became eligible for care under the LTCI during the 10-year follow-up. In this system, certification of long-term care needs was based on an evaluation of each applicant’s degree of physical and mental disability, determined by a home-visit interview and a diagnosis from the primary care physician. A municipality certification committee determines the eligibility for receiving services [21].

### 2.3. Explanatory Variables

To elucidate social ties in this study, we asked respondents about five types of social support with persons in the three social network categories of co-residing family, family and relatives living apart, and friends and neighbors. “Family” refers to a spouse or partner, children, siblings, older parents, and other relatives. Types of support were emotional (providing/receiving), instrumental (providing/receiving), and appraisal (receiving). “Listening to concerns and complaints” was regarded as emotional support and “looking after when sick in bed for a few days” as instrumental support. Appraisal support was elicited by asking, “Do you have someone who acknowledges your existence and value?”

We considered that respondents had social ties when at least one person was giving or receiving any of above five types of support in specific network category. Social ties in each network category were then dichotomized as “having ties” (coded 1) or “not having ties” (coded 0). Our main aim was to study the relative effect of social ties in three different social network domains. Thus, we dichotomized social ties in each network category to calculate the relative risk of disability. In other words, we wanted to know what happens to a person with no ties, exchanging no support, with family members.

### 2.4. Covariates

Controlling variables were age in years, health status, and living arrangement. Age and health status are important confounders when assessing the relationship between social ties and incident disability [1,22]. Health status was elicited by asking whether the participant was under medical treatment or not. We asked, “Are you currently receiving any medical treatment?”. The answering categories were, “I have no illnesses or conditions”, “I have illnesses or conditions but need no treatment”, “I discontinued treatment on my own decision”, and “I am currently receiving medical treatment”. Living arrangement in five categories, alone, only with spouse, with spouse and children, no spouse with children, other, was treated as a covariate. In sub-analyses, we analyzed the data by stratifying those living alone and those living with someone. The direction of the association between social ties and functional decline was the same. Moreover, in our sample, 6.5% of men and 10.9% of women did not have anyone with whom to exchange support despite living in the same house. Thus, instead of analyzing data by stratifying those living alone and those living with someone else, we treated living arrangement as it was, as studies suggested that loneliness has a stronger effect on health [1,23]. Our underlying assumption was that being isolated psychologically rather than living alone, per se, had higher risk for functional decline.

### 2.5. Statistical Analyses

Age-adjusted cumulative incidence of functional decline was calculated using a general linear model for each covariate. A Cox hazard proportional model stratified by sex was employed to calculate hazard ratios for functional decline since sex differences were observed in the associations of social relations and health in previous studies [1,2,3]. Those who died or moved away from the study site during the follow-up period were considered as censored cases.

To test whether the effects of each factor were independent from the influence of the others, we used hierarchical regression modeling procedures. First, we constructed a model adjusted for age in years and living arrangement to predict incident disability. Then, we added health status in the second model to consider the effect of ill health on need-driven cohabitation. “Need-driven” means those who need support due to ill health are more likely to live with family members, especially with adult children [16]. Finally, we entered all social ties in the three network categories simultaneously along with age, living arrangement, and health status to evaluate the independent effect of each social tie.

We used SPSS 21.0J (SPSS, Chicago, IL, USA) for statistical analysis. A *p*-value of less than 0.10 was considered marginally significant, and a *p*-value less than 0.05 was considered statistically significant.

## 3. Results

Table 1 represents baseline characteristics of respondents by gender. Descriptive data analysis was conducted to assess gender differences. *P*-values were calculated using chi-squared tests for categorical data and *t*-tests for continuous data. Men were slightly younger and healthier than women. Although more men lived with someone else, they had fewer ties with someone outside of their own home.

Table 2 represents the age-adjusted cumulative incidence of disability during the 10-year follow-up period. Those who lacked social ties (e.g., giving or receiving any five types of social support) in each network category had higher incidence of disability compared to those who with such ties.

Table 3 shows the results of the multivariate Cox proportional hazard models. In Model 3, presence or absence of social ties in each of the three network domains was entered simultaneously to evaluate the relative effect of each tie on disability onset. We found that social ties with co-residing family members and ties with friends or neighbors protected men’s functional health with hazard ratios of 0.81 and 0.85, respectively. Men with no social ties with co-habitants but with friend and neighbor ties, had a 15% reduction in disability risk, while such risk reduction was approximately 31% for counterparts with both co-residing family ties and friend or neighbor ties. Among women, ties with friend or neighbors had significant effect on health with a hazard ratio of 0.89. Women with friends or neighbor ties had an 11% lower risk compared to those without. On the contrary, support exchange with family or relatives living apart had no significant effect on maintenance of functional health both among men and women.

## 4. Discussion

### 4.1. Different Effects of Support Exchange by Different Support Network Members

Using follow-up data from almost 10 years’ time, we assessed the effect of social ties in different network categories on disability onset, defined as dependence for basic activities of daily living, and found that ties with family members living together were beneficial for men even after controlling for ties with other network members. Among women, this effect disappeared when ties with other sources were considered in the model. Among them, ties with friends or neighbors were stronger predictors of maintenance of functional health. This is in line with the findings in other studies that men tend to report their spouses as confidants whereas women do not [1,22]. It is possible that, due to their longer lifespan, women are more likely to be widowed and cannot rely on their spouses in case of need. In our sample, ties with family or relatives living apart had no significant effect on functional health among either men or women. This might be that the availability of support from family, which was mostly adult children, or relatives living apart might indicate the existence of care needs among older persons. In other words, those already in ill health might have been included from the group, although we adjusted for health status in the model. Geographical proximity of family or relatives living apart or frequency of contact might also explain the result. In addition, support from family members is often accompanied with obligation, which may not always be a positive influence, while support from friends is mostly voluntary and characterized by activities of common interest [1,22]. Moreover, to interact with non-family persons, one must leave the home. This might lead to receiving more physical and cognitive stimuli in daily life to maintain functional health.

In addition, the replacement function of social ties might also explain the result. A study in China demonstrated the importance of extended family in the well-being of older persons, especially when support from immediate kin was not available [12]. This explains the findings that a person with diverse network types had better health compared to those with restricted networks [12]. In our study, those living alone had more contact with outside family members [9]. When support functions become inadequate or are no longer available, the person tends to create new ties to substitute for lost ones [1]. Those lacking family ties might try to meet their social needs by increasing interactions with non-family members. A study in England demonstrated that weaker ties, relative to more bonding relationships, were beneficial because these were based on reciprocity and did not implicate burden or stigma about receiving support [23].

### 4.2. The Role of Social Connectedness

Studies indicated that social connectedness, measured by the number of organizations belonged to or the frequency of contacts with family, relatives, and friends or neighbors, was beneficial for health [1,7]. In other studies using the AGES project data, older people who exercised in groups were less likely to face disability [4,5], indicating how health is protected by having company during activities. Possible pathways linking group exercise to better functional health were psychological (i.e., enjoyment, encouragement) as well as social effects (i.e., social support, social influence) [4,5].

For older persons to continue living in a community, having someone to exchange support, irrespective of living arrangement, seemed to be important. In this study, those with no ties at all accounted for about 5% of the total sample (5.2% for men and 5.0% for women). As shown in Table 2, those with no ties at all had more risk of disability compared to those with at least one type of social tie. The age-adjusted cumulative incidence of functional decline was 29.6% for men and 27.7% for women with no ties at all. Before the LTCI system, care for older people with disabilities was mainly provided by family members. Clearly, those living alone are at a disadvantage in terms of support availability. Given the fact that the number of older people living alone is increasing [16,18], promoting social ties with outside family members might be promising for safe living and independence in the community among older persons. In this sense, our study has several implications for future practices. Conventionally, many community programs for older persons target those living alone. However, in sub-analysis, 10.3% of men and 23.6% of women had no ties at all, not having any five of the social support types, with the members living in the same house (data available upon request). In other words, they were isolated psychologically although living with someone else. This implies the importance of including all people with fewer social ties irrespective of living arrangement.

### 4.3. Strengths and Limitations

The major strength of our study is that we used insurance data maintained by municipalities with very few missing cases. Use of insurance data enables us to better estimate factors associated with the onset of functional disability. The present study adds several new findings to those of earlier studies. First, a longitudinal effect of social ties, assessed by social support exchange, on functional health was observed. Second, a protective effect of social ties with non-family members on functional health was independent of family ties. In other words, those with no family ties may also receive benefits by exchanging support with someone outside family, such as friends or neighbors. Third, the relative advantages of having ties with non-family members for functional health were observed among women. This is important, since the number of women living alone is increasing due to their longer lifespan [16].

One limitation of this study is that we used a rough measure of social ties by only using five types of social support and three network domains. Detailed measurement of social ties might produce more reliable results. Another limitation is that we could not distinguish social exchange members in each network domain. For example, in our sample, it was impossible to know if they were spouses or children or other members, since we did not ask with whom they exchanged support. As studies have indicated, ties with spouses are more important for older individuals’ well-being [10]; this might confound the effect of ties with spouses and other family members. In addition, we only measured the baseline living arrangement. As living arrangements may change over time, this might have confounded the association. In our cohort, 482 cases (3.4%) among 14,088 persons relocated during the almost 10 years of follow-up. Although, in Japan, living arrangements are quite stable over time due to a lower rate of needs-driven cohabitation [16,17], we need to be cautious when interpreting the result.

Lastly, 8% of older persons did not provide information on their activities of daily living (bathing, using the toilet, or transferring), nor did they apply for the LTCI services despite being dependent for basic activities of daily living at the baseline. Although we excluded those persons to minimize possible bias, identifying those who developed disability during the follow-up was impossible, unless they applied for the LTCI services and were included in the database. This will need to be addressed in future studies.

## 5. Conclusions

Social ties protected older persons from functional decline. The fact that social ties with friends or neighbors were associated with a lower risk of functional decline even in the absence of family support, both among men and women, serves to underscore the importance of social ties, especially among those living alone or those having no such ties with co-residing family members. For the health of older persons, the role of social support needs to be reconsidered. Further studies using more sophisticated measures are needed to clarify the longitudinal effect of social ties on health, especially the effect of support exchange with non-family members. In addition, social intervention to promote friendship and social activities might be promising for the maintenance of an older person’s functional ability.

## Figures and Tables

**Table 1:** Baseline characteristics of the study population.

	Mean ± SD or *N* (%)				*p*-Values
	Men *N* = 6906		Women *N* = 7182		
Age in years (65–99)	72.3	±5.63	73.1	±6.10	*p* < 0.000
Health status					
No illnesses/conditions	1252	(18.1)	1148	(16.0)	*p* < 0.000
With illnesses/conditions but need no treatment	789	(11.4)	585	(8.1)	
With illnesses/conditions but discontinued treatment	404	(5.8)	471	(6.6)	
Under medical treatment	4233	(61.3)	4586	(63.9)	
Missing	228	(3.3)	392	(5.5)	
Living arrangement					
Alone	287	(4.2)	1098	(15.3)	*p* < 0.000
Only with spouse	3065	(44.4)	2053	(28.6)	
With spouse and with children	2397	(34.7)	1505	(21.0)	
Without spouse and with children	464	(6.7)	1683	(23.4)	
Other	568	(8.2)	651	(9.1)	
Missing	125	(1.8)	192	(2.7)	
Social ties ^a^					
Co-residing family (Yes)	6195	(89.7)	5484	(76.4)	*p* < 0.000
Family/relative living apart (Yes)	3394	(49.1)	4552	(63.4)	*p* < 0.000
Friends/neighbors (Yes)	4238	(38.6)	3940	(54.9)	*p* < 0.000
No ties at all ^b^	362	(5.2)	360	(5.0)	n.s.

^a^ Social ties in each network category was recognized when at least one person in that category was giving or receiving any one of the three kinds of support: emotional, instrumental, or appraisal; ^b^ This refers to someone reporting no one to give or receive any one of the three kinds of support irrespective of network categories. *p*-values in the table are for gender differences.

**Table 2:** Age-adjusted rate of the cumulative incidence of disability during follow-up by social ties.

		*N*	Incidence Disability	% (Age-Adjusted %) ^a^	*N*	Incidence Disability	% (Age-Adjusted %) ^a^
	Men (*N* = 6906)				Women (*N* = 7182)		
Co-residing family	Yes	6195	1324	21.4 (22.8)	5484	1338	24.4 (24.0)
	No	711	219	30.8 (26.0)	1698	498	29.3 (27.9)
Family living apart	Yes	3394	767	22.6 (25.1)	4552	1108	24.3 (25.0)
	No	3512	776	22.1 (23.7)	2630	728	27.7 (26.9)
Friends/neighbors	Yes	2668	512	19.2 (22.9)	3940	881	22.4 (25.5)
	No	4238	1031	24.3 (25.9)	3242	955	29.5 (26.4)
All ties combined	Yes	6544	1421	21.7 (23.7)	6822	1714	25.1 (25.7)
	None	362	122	33.7 (29.6)	360	122	33.9 (27.7)

Figures in the table are the number of cases unless otherwise specified. **^a^** Figures in parentheses are percentages adjusted for mean age (72.7) using a general linear model.

**Table 3:** Hazard ratios for incident disability using a Cox proportional hazard model.

	Model 1	Model 2	Model 3
	HR (95% CI)	HR (95% CI)	HR (95% CI)
Men (*N* = 6906)			
Co-residing family (Yes)	0.81 (0.67–0.97) *	0.79 (0.65–0.95) *	0.81 (0.67–0.98) *
Family/relative living apart (Yes)	0.98 (0.88–1.08)	0.97 (0.87–1.07)	1.02 (0.91–1.14)
Friends/neighbors (Yes)	0.86 (0.77–0.95) **	0.85 (0.76–0.95) **	0.85 (0.76–0.96) **
Women (*N* = 7182)			
Co-residing family (Yes)	0.87 (0.75–1.01) ^†^	0.87 (0.75–1.06) ^†^	0.89 (0.76–1.04)
Family/relative living apart (Yes)	0.98 (0.87–1.08)	0.94 (0.85–1.04)	0.97 (0.87–1.07)
Friends/neighbors (Yes)	0.88 (0.80–0.96) **	0.88 (0.79–0.97) **	0.89 (0.80–0.98) *

Model 1: adjusted for age, and living arrangement; Model 2: adjusted for age, living arrangement, and health status; Model 3: adjusted for age, living arrangement, health status. Presence or absence of social ties in each network domain (co-residing family, family living apart, and friends/neighbors) was mutually adjusted by being entered simultaneously in the Model 3. Reference categories in Cox proportional hazard models are “No” (absence of social ties) for each network category. ^†^ *p* < 0.10, * *p* < 0.05, ** *p* < 0.01.
